# Supplementary material for: High-throughput screen reveals sRNAs regulating crRNA biogenesis by targeting CRISPR leader to repress Rho termination
Source: Nat Commun. 2019 Aug 19;10:3728. doi: 10.1038/s41467-019-11695-8 (PMC6700203; doi:10.1038/s41467-019-11695-8)
Supplement: Supplementary file 1 — Supplementary Information [file 41467_2019_11695_MOESM1_ESM.pdf]

# Supplementary information

**High-throughput screens reveal sRNAs regulating crRNA biogenesis by targeting CRISPR leader to repress Rho termination**

**Authors:** Ping Lin, Qinqin Pu, Qun Wu, Chuanmin Zhou, Biao Wang, Jacob Schettler, Zhihan Wang, Shugang Qin, Pan Gao, Rongpeng Li, Guoping Li, Zhenyu Cheng, Lefu Lan, Jianxin Jiang, Min Wu

Correspondence to: Min Wu, min.wu@med.und.edu; Jianxin Jiang, hellojjx@126.com; Lefu Lan, llan@simm.ac.cn.

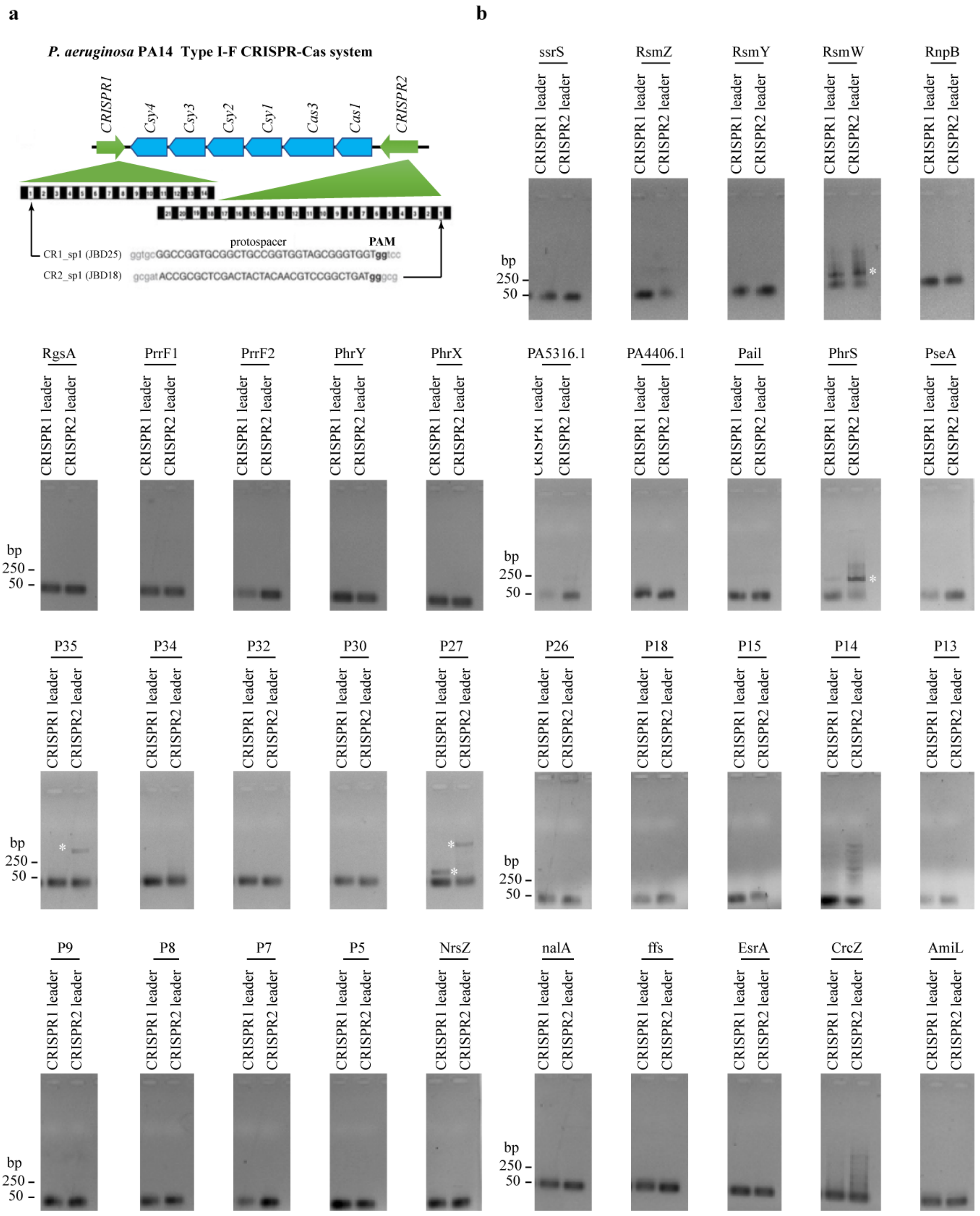


**Supplementary Figure 1. Detection of sRNA binding to *P. aeruginosa* type I-F CRISPR-Cas system. (a)** Schematic of the PA14 CRISPR loci and cas gene regions. The DNA sequences of the protospacers tested for CRISPR-target Plasmids (CR1-sp1, CR2-sp1) and phages (JBD18, JBD25). **(b)** Detection of sRNA-containing chimaeras for determination of sRNAs linking to CRISPR leader in vivo. The white stars represent the amplicons for sRNA-CRISPR leader chimaeras. PCR amplification were used the primers for target sRNAs and CRISPR leader.

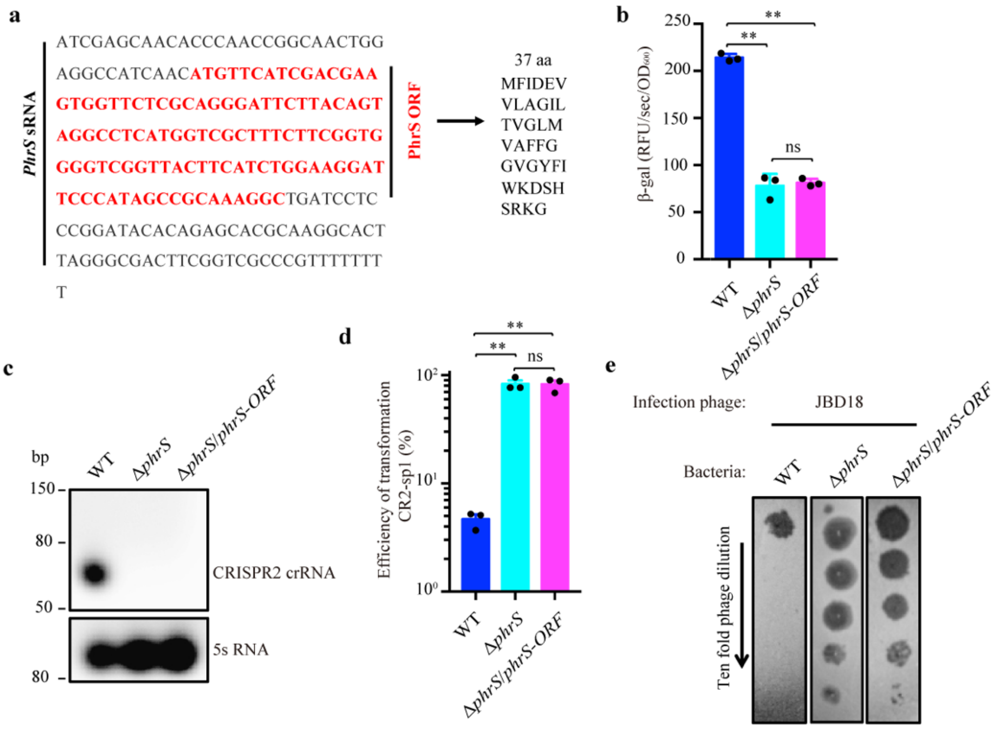


**Supplementary Figure 2. An ORF in PhrS sRNA has no effect on CRISPR-Cas adaptive immunity. (a)** Red sequences show the translational start and stop codons of the PhrS encoded ORF. **(b)** *CRISPR2-lacZ* activity in the PA14 WT, Δ*phrS*, Δ*phrS/phrS-ORF*. **(c)** Northern-blot analysis of CRISPR2 crRNA levels in PA14 Δ*phrS* background strain containing the internal ORF of PhrS. **(d)** Transformation efficiency of CRISPR-targeted plasmids CR2-sp1 in the PA14 WT or Δ*phrS* mutant. **(e)** Tenfold dilutions of lysates of a CRISPR-sensitive phage JBD18 were applied to bacterial lawns of PA14 Δ*phrS* background strain with the internal ORF of PhrS. Results are presented as mean ± SEM from three independent experiments. Statistical significance was assessed by one-way ANOVA plus Tukey test (**P<0.01; *P<0.05).

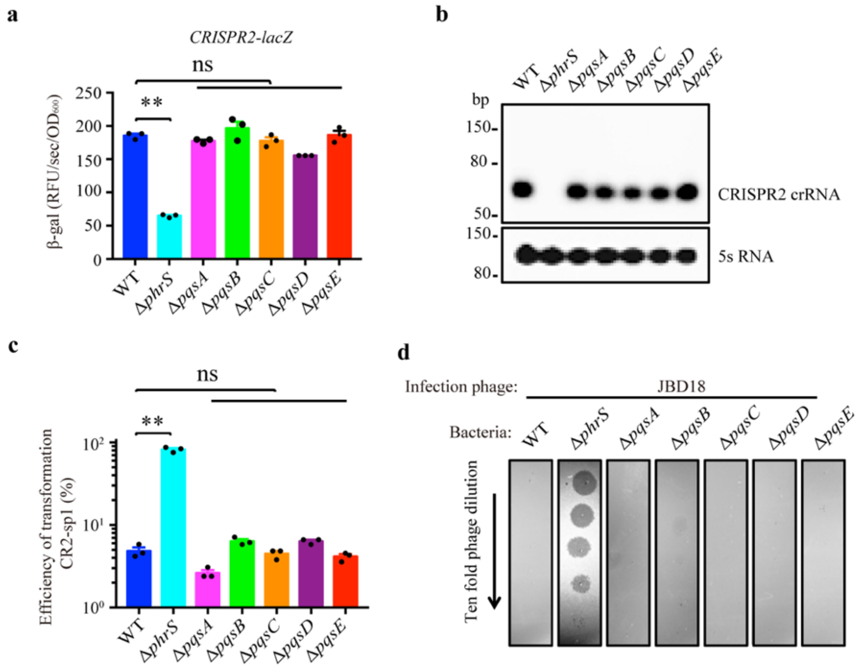


**Supplementary Figure 3. PqsA-E has no effect on regulation of CRISPR-Cas adaptive immunity. (a)** *Cas-lacZ* and *CRISPR-lacZ* activity for the type I-F reporter strains in the *PqsA-E* mutant backgrounds. **(b)** Northern-blot of CRISPR2 crRNA production in the *PqsA-E* mutant backgrounds. **(c)** Transformation efficiency of CRISPR-targeted plasmids in the *PqsA-E* mutant backgrounds. **(d)** Tenfold dilutions of lysates of a CRISPR-sensitive phage JBD18 were applied to bacterial lawns of the *PqsA-E* mutant backgrounds. Results are presented as mean ± SEM from three independent experiments. Statistical significance was assessed by one-way ANOVA plus Tukey test (**P<0.01; *P<0.05).

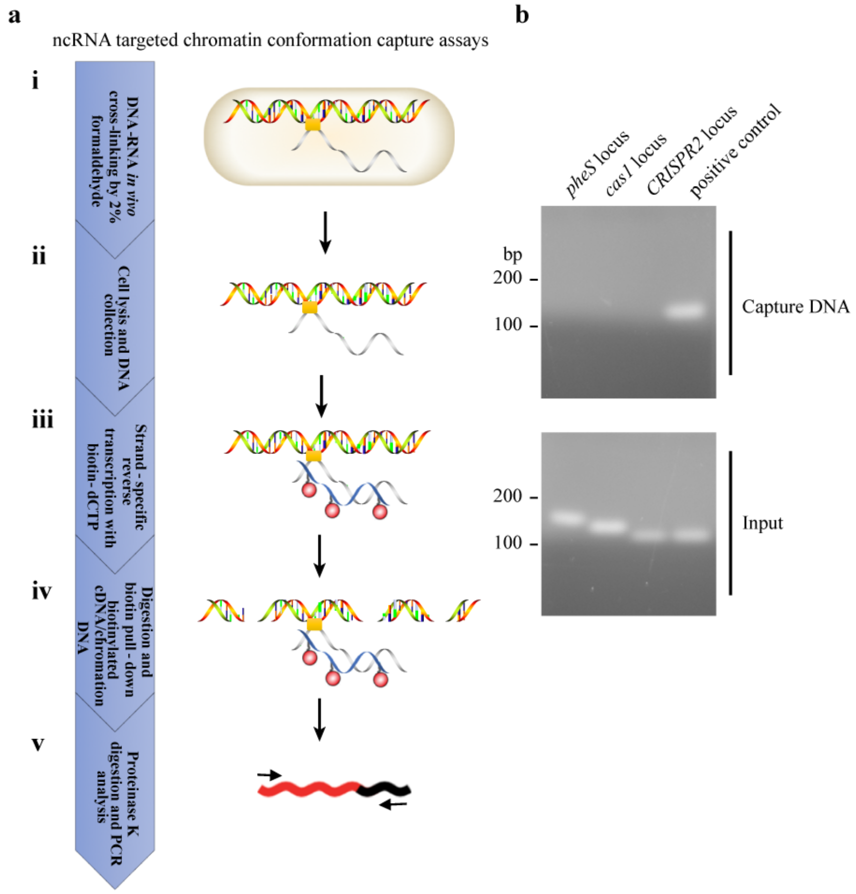


**Supplementary Figure 4. Chromatin DNA has no interaction with PhrS. (a)** The diagram for detection of PhrS sRNA-DNA interaction. A reverse transcription-associated trap (RAT) assay was used to detect the PhrS-specific interaction with CRISPR locus DNAs, including (i) DNA-RNA cross-linking by 2% formaldehyde, (ii) cell lysis and bacterial chromosomal DNA collection, (iii) strand-specific reverse transcription with biotin-dCTP, (iv) digestion and biotin poll-down biotinylated cDNA/chromosomal DNA, and (v) proteinase K digestion and PCR analysis. **(b)** PCR detection of PhrS-DNA interaction in PA14 strain. Note no enriched binding of PhrS at the DNA CRISPR2 locus. Input: RNA-chromatin complex aliquots collected before RAT. The positive control is the *IRAIN* lncRNA binding to chromatin DNA.

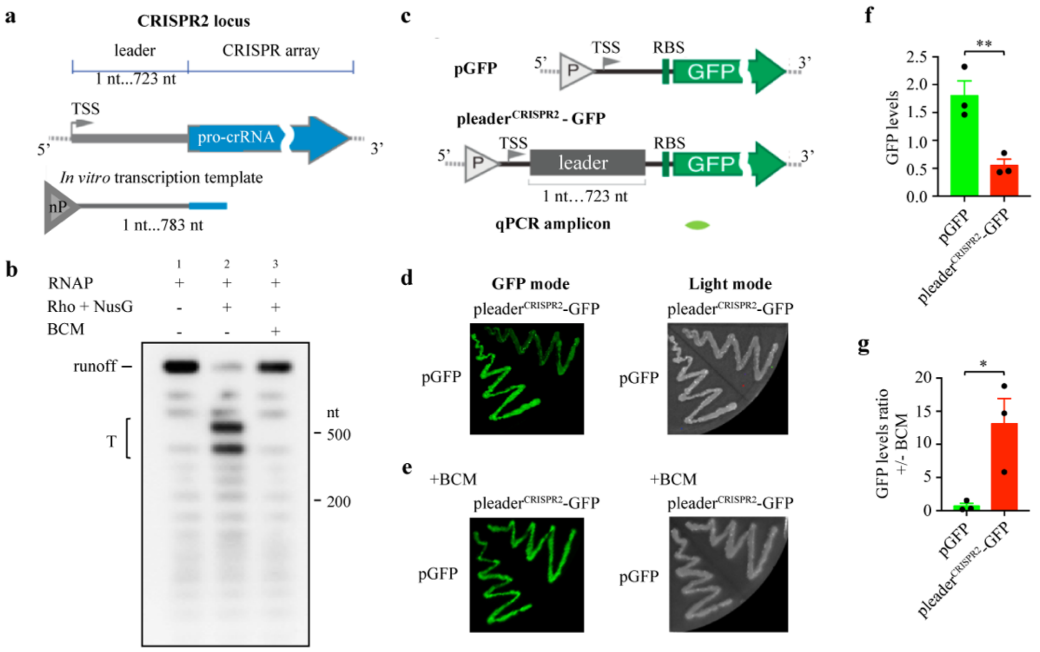


**Supplementary Figure 5. Rho-dependent termination within CRISPR2 leader sequence. (a)** A diagram of PA14 CRISPR leader sequence and pre-crRNA indicated. A genome-derived template for in vitro transcription (b) that contains the CRISPR2 leader followed by 60 bp first repeat with spacer sequence. **(b)** A representative single round transcription on the CRISPR2 locus template (see a). Preformed elongation complexes were chased without (lane 1) or with Rho and NusG (without bicyclomycin (BCM), lane 2; with BCM, lane 3). The most prominent termination products are marked with bracket. The runoff is indicated. **(c)** Reporter constructs for pGFP and pleader$^{CRISPR}$-GFP. Upper: pGFP construct with the GFP reporter only. Lower: the transcriptional fusion pleader$^{CRISPR}$-GFP used to test the effect of the CRISPR leader on Rho termination. "P" indicates the location of a constitutive promoter. TSS represents the transcription start site; RBS indicates the ribosome-binding site. The location of qRT-PCR amplicon is indicated below (green). **(d)** Representative results from the GFP plate assay. PA14 WT transformed with pGFP and pleader$^{CRISPR}$-GFP grew on LB agar plates. The fluorescence intensity was measured (GFP mode, left). The same plate was also captured under visible light (light mode, right). **(e)** Representative results from the GFP plate assay. PA14 WT transformed with pGFP and pleader$^{CRISPR}$-GFP grew on LB agar plates with 8 ug/ml BCM. The fluorescence intensity was measured (GFP mode, left). The same plate was also captured under visible light (light mode, right). **(f)** qRT-PCR data for GFP expression in PA14 WT transformed with pGFP and pleader$^{CRISPR}$-GFP. **(g)** Ratio for GFP expression in PA14 WT transformed with pGFP and pleader$^{CRISPR}$-GFP within or without BCM. Results are presented as mean ± SEM from three independent experiments. Statistical significance was assessed by one-way ANOVA plus Tukey test (**P<0.01; *P<0.05).

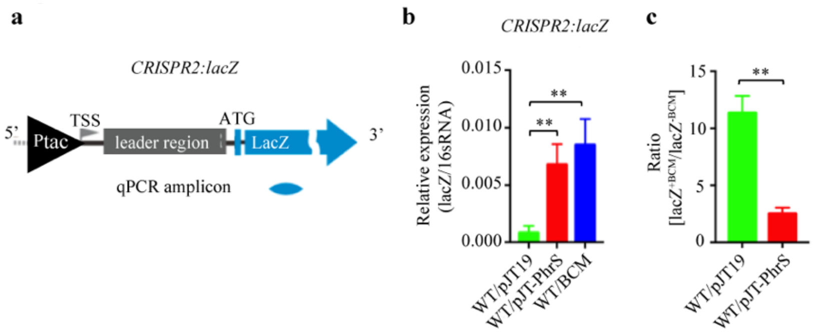


**Supplementary Figure 6. PhrS overexpression stimulates CRISPR2-driven transcription by inhibiting Rho. (a)** The CRISPR2:lacZ chromosomal reporter fusion with the inducible Ptac promoter. **(b)** Transcription levels of *lacZ* measured in WT strain carrying CRISPR2:lacZ chromosomal reporter fusion. Where indicated the expression of plasmid-borne PhrS sRNA. **(c)** The effect of BCM on *lacZ* transcript levels in the strains overexpression PhrS. Results are presented as mean ± SEM from three independent experiments. Statistical significance was assessed by one-way ANOVA plus Tukey test (**P<0.01; *P<0.05).

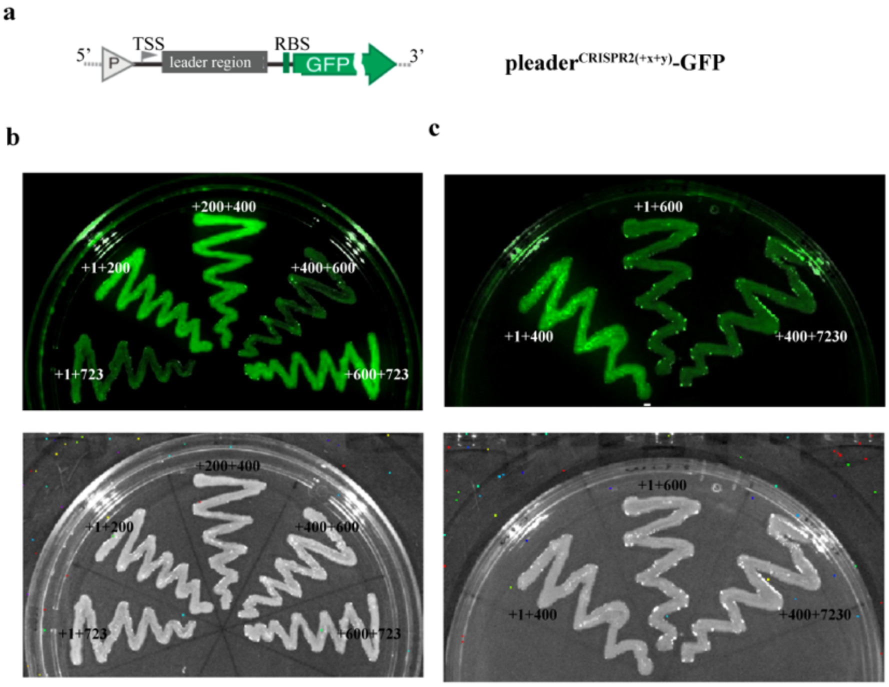


**Supplementary Figure 7. PhrS controls Rho-dependent termination of CRISPR-Cas system by binding to CRISPR leader sequence within a +400~+600 segment. (a)** A schematic of the transcriptional reporter fusion pleader$^{CRISPR(+x+y)}$-GFP used to test the effect of different fragments of the leader sequence on Rho termination. +x+y coordinates indicate the beginning and the end of the test leader fragment, counting from the TSS. **(b)** Representative results from GFP plate assay. PA14 strain transformed with pleader$^{CRISPR(+x+y)}$-GFP plasmid grew on LB agar plates and the fluorescence intensity was measured (GFP mode, upper). The same plates were also captured under visible light (Light mode, lower). **(c)** Representative results from GFP plate assay. PA14 strain transformed with pleader$^{CRISPR(+x+y)}$-GFP plasmid grew on LB agar plates and the fluorescence intensity was measured (GFP mode, upper). The same plates were also captured under visible light (Light mode, lower).

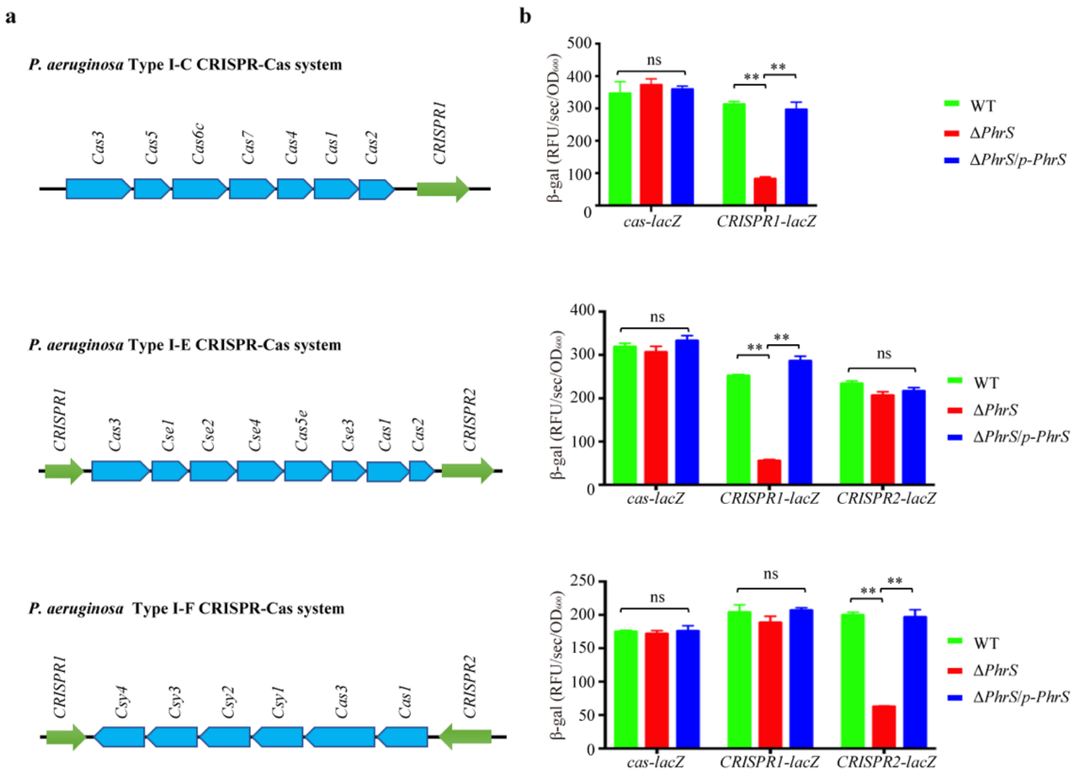


**Supplementary Figure 8. PhrS regulates expression of CRISPR loci in three distinct CRISPR-Cas systems.** **(a)** Schematic of *P. aeruginosa* ST277, SM4386, and PA14, which possess type I-C, I-E, and I-F CRISPR-Cas systems, respectively. Genes encoding interference or adaptation machinery are colored blue. The CRISPR loci are colored green. **(b)** *cas-lacZ* and *CRISPR-lacZ* activity for each of type I-C, type I-E, and type I-F reporter strains in the WT and PhrS mutant backgrounds. Results are presented as mean ± SEM from three independent experiments. **P<0.01, *P<0.05, one-way ANOVA plus Tukey test.

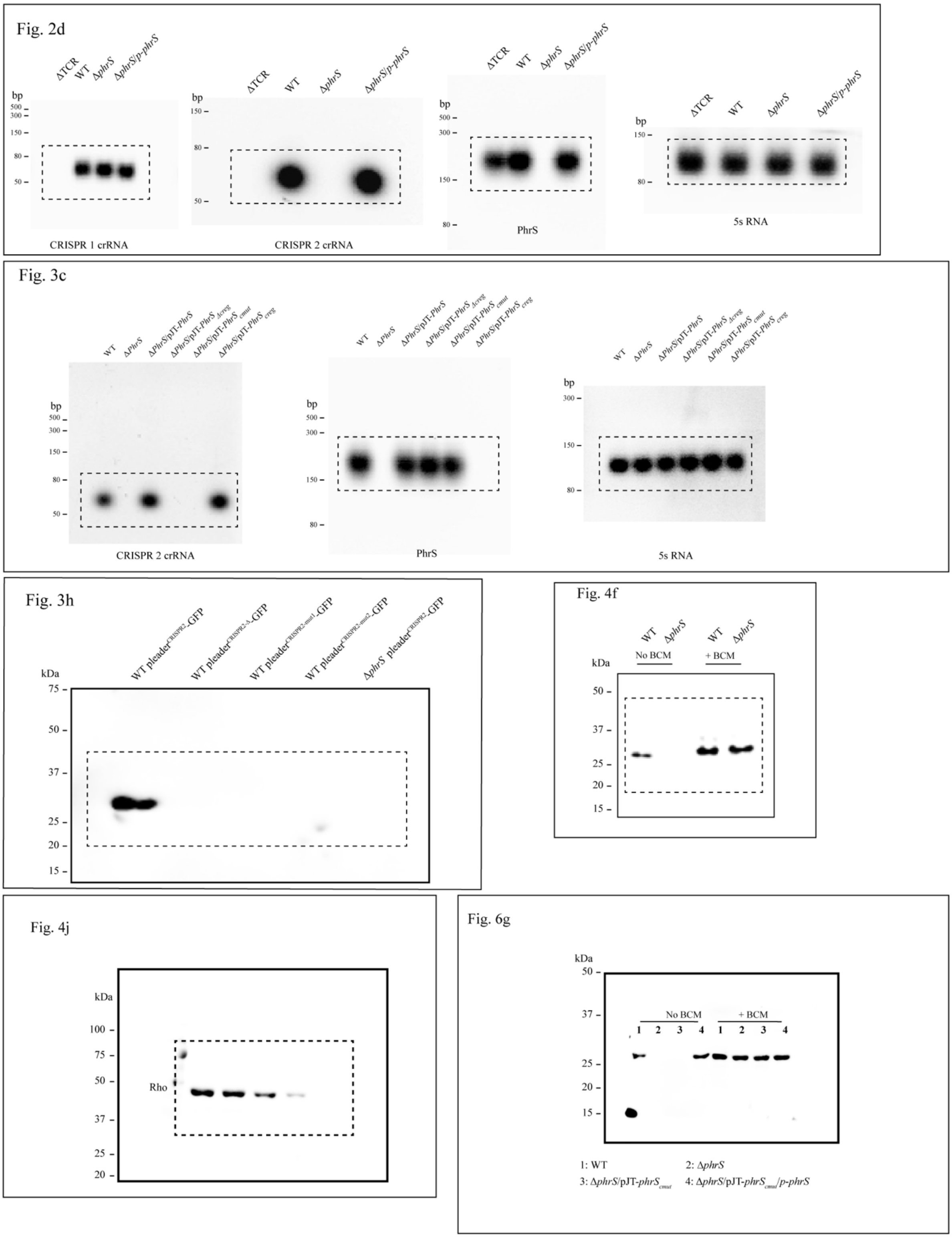


**Supplementary Figure 9. Uncropped blots and gels of Fig. 2d, 3c, 3h, 4f, 4j and 6g.**

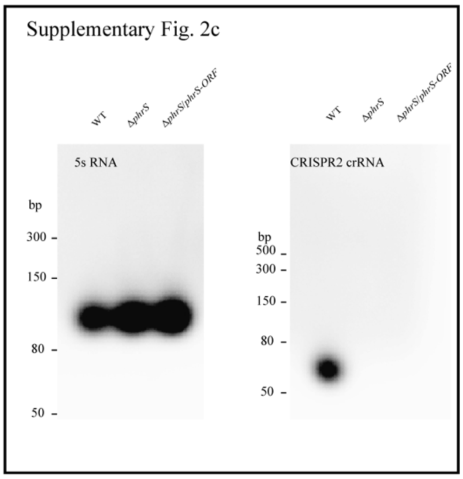


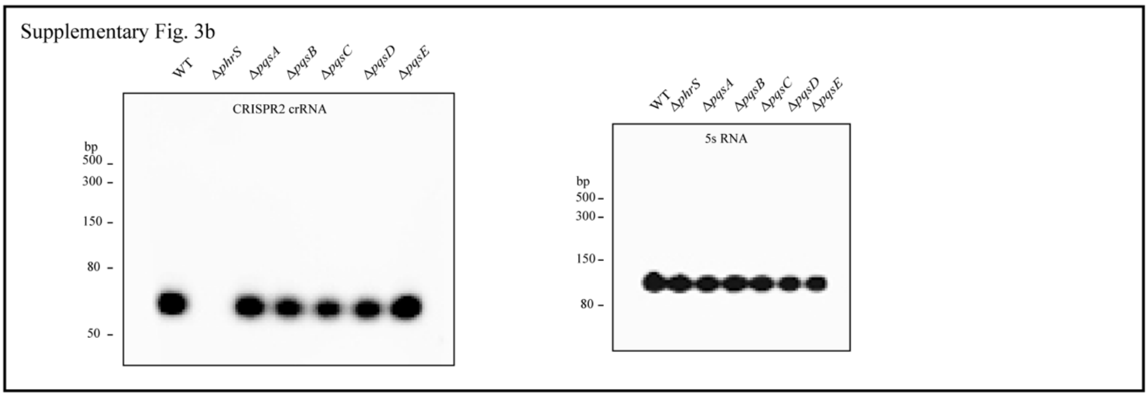


**Supplementary Figure 10. Uncropped blots and gels of Supplementary Fig. 2c and 3b.**

**Supplementary Table 1. Oligonucleotides used in this study**.

| Name | sequences |
|---|---|
| PhrS-probe | TAACCGACCCCACCGAAGAAAGCGACCATGAGGC |
| 5S rRNA-probe | CCCCACACTACCATCGGCGATGCGTCG |
| CRISPR1 crRNA-probe | GGCCGGTGCGGCTGCCGGTGGTAGCGGGTGGT |
| CRISPR2 crRNA-probe | ACCGCGCTCGACTACTACAACGTCCGGCTGAT |
| GFP-F | TGGAGAGGGTGAAGGTGAT |
| GFP-R | AGCATTGAACACCATAAGTCAAAG |
| CRISPR2 UTR-F | TCCAACCCCGAGGTAACAGA |
| CRISPR2 UTR R | TCTCATCGAGCAAACGGGTC |
| CRISPR2 5’ORF-F | CTGGCGGGAAAAACTCGGTA |
| CRISPR2 5’ORF-R | ACGGCAGTGAACTTGACGAA |
| CRISPR2 ORF-F | CAGCATCGAGACGGTTCACT |
| CRISPR2 ORF-R | CCGGGCTGTTCGAGGATTTT |
| lacZ 5’ORF-F | AAAACCCTGGCGTTACCCAA |
| lacZ 5’ORF-R | CCTCAGGAAGATCGCACTCC |
| lacZ ORF-F | AAACCTCAGTGTGACGCTCC |
| lacZ ORF-R | TGGCGGTTAAATTGCCAACG |

## Supplementary Table 2. Templates for in vitro transcription.

| | |
|---|---|
| CRISPR2 template with native promoter (1 nt…783 nt, from TSS-shown in bold, repeat sequence shown in red, spacer 1 shown in blue) | TACAGGCCCTGGAAACGCCGGACAGCTCCCCCGGACATTTCCGCAGGCTCTCTCAGAAGCCCGG<br>GAATTGGATAAAAACCCAGTAGGAGGATGATGGAGG**GGCCGGCAAATCCATCCCGCAGGGAC**<br>**AGGACCGAACGGCACTGCCCTCTATTTTCTGAGGGCAAAAAAGACGCGGGCCGCAGGTG**<br>**GTGGAACACCTACGACCCGCTAACCACAAACCAGCTTATGAAGGAGCTGACCCATGGCTA**<br>**AGCCGAATGATACGTCCAACCCCGAGGTAACAGAATCGTCCCCCCGCTTCGTCCCGCTGC**<br>**TCCCCACCTCCCAGGCCGACATGTGTCCGCGTAGCGCCACCCTGTACGTGGACACCCATG**<br>**CACTCCCCACGCACTTGTTCGACAACGCGAAAGTGCGCTTGAGGTCGGCGATCAACATCC**<br>**TGGCCATGACCCGTTTGCTCGATGAGAAAGAAGAGCCCGGCGGCACCCTGGCCTACATCA**<br>**ACGGTGCGTTGCTGTCGCTGCTCAGCGATGCCTTGTCCGAAATCGAGGCGGGCCACCCGA**<br>**GCCTGTAGCGCCGCTCCCTGGCGCGACGCCCCCGGCCTGGCCGGGGGCTTTGCGGCATC**<br>**GCCCATCAAAAGACCTTTCGCGCCCGAACGGCACGGTTGATCGCCGTCCCGGTCCTCGCG**<br>**AAACGGCCGCCAATTGCCCGAAGCTTCCGACCCTTTTTTCGGACGATTTCTTACGCCCTTA**<br>**TAAATCAGCAAGTTACGAGACCTCGAAAAAAGAGGGTTTCTGGCGGGAAAAACTCGGTAT**<br>**TTCTTTTTCCTTCAAATGGTTATAGGTTTTCGGAGCTA**GTTCACTGCCGTGTAGGCAGCTAAG<br>AAAATCAGCCGGACGTTGTAGTAGTCGAGCGCGGT |
| CRISPR2-Δ template with native promoter (1 nt…772 nt, from TSS-shown in bold, repeat sequence shown in red, spacer 1 shown in blue, 11 nucleotides deletion shown in dot) | TACAGGCCCTGGAAACGCCGGACAGCTCCCCCGGACATTTCCGCAGGCTCTCTCAGAAGCCCGG<br>GAATTGGATAAAAACCCAGTAGGAGGATGATGGAGG**GGCCGGCAAATCCATCCCGCAGGGAC**<br>**AGGACCGAACGGCACTGCCCTCTATTTTCTGAGGGCAAAAAAGACGCGGGCCGCAGGTG**<br>**GTGGAACACCTACGACCCGCTAACCACAAACCAGCTTATGAAGGAGCTGACCCATGGCTA**<br>**AGCCGAATGATACGTCCAACCCCGAGGTAACAGAATCGTCCCCCCGCTTCGTCCCGCTGC**<br>**TCCCCACCTCCCAGGCCGACATGTGTCCGCGTAGCGCCACCCTGTACGTGGACACCCATG**<br>**CACTCCCCACGCACTTGTTCGACAACGCGAAAGTGCGCTTGAGGTCGGCGATCAACATCC**<br>**TGGCCATGACCCGTTTGCTCGATGAGAAAGAAGAGCCCGGCGGCACCCTGGCCTACATCA**<br>**ACGGTGCGTTGCTGTCGCTGCTCAGCGATGCCTTGTCCGAAATCGAGGCGGGCCACCCGA**<br>**GCCTGTAGCGCCGCTCCCTGGCGCGACGCCCCCGGCCTGGCCGGG..........ATCGCCCATCA**<br>**AAAGACCTTTCGCGCCCGAACGGCACGGTTGATCGCCGTCCCGGTCCTCGCGAAACGGC**<br>**CGCCAATTGCCCGAAGCTTCCGACCCTTTTTTCGGACGATTTCTTACGCCCTTATAAATCA**<br>**GCAAGTTACGAGACCTCGAAAAAAGAGGGTTTCTGGCGGGAAAAACTCGGTATTTCTTTT**<br>**TCCTTCAAATGGTTATAGGTTTTCGGAGCTA**GTTCACTGCCGTGTAGGCAGCTAAGAAAATCA<br>GCCGGACGTTGTAGTAGTCGAGCGCGGT |
| CRISPR2-mut1 template with native promoter (1 nt…783 nt, from TSS-shown in bold, repeat sequence shown in red, spacer 1 shown in blue, 2 point nucleotides mutation shown in green) | TACAGGCCCTGGAAACGCCGGACAGCTCCCCCGGACATTTCCGCAGGCTCTCTCAGAAGCCCGG<br>GAATTGGATAAAAACCCAGTAGGAGGATGATGGAGG**GGCCGGCAAATCCATCCCGCAGGGAC**<br>**AGGACCGAACGGCACTGCCCTCTATTTTCTGAGGGCAAAAAAGACGCGGGCCGCAGGTG**<br>**GTGGAACACCTACGACCCGCTAACCACAAACCAGCTTATGAAGGAGCTGACCCATGGCTA**<br>**AGCCGAATGATACGTCCAACCCCGAGGTAACAGAATCGTCCCCCCGCTTCGTCCCGCTGC**<br>**TCCCCACCTCCCAGGCCGACATGTGTCCGCGTAGCGCCACCCTGTACGTGGACACCCATG**<br>**CACTCCCCACGCACTTGTTCGACAACGCGAAAGTGCGCTTGAGGTCGGCGATCAACATCC**<br>**TGGCCATGACCCGTTTGCTCGATGAGAAAGAAGAGCCCGGCGGCACCCTGGCCTACATCA**<br>**ACGGTGCGTTGCTGTCGCTGCTCAGCGATGCCTTGTCCGAAATCGAGGCGGGCCACCCGA**<br>**GCCTGTAGCGCCGCTCCCTGGCGCGACGCCCCCGGCCTGGCCGGGGGCTAAGCGGCATC**<br>**GCCCATCAAAAGACCTTTCGCGCCCGAACGGCACGGTTGATCGCCGTCCCGGTCCTCGCG**<br>**AAACGGCCGCCAATTGCCCGAAGCTTCCGACCCTTTTTTCGGACGATTTCTTACGCCCTTA**<br>**TAAATCAGCAAGTTACGAGACCTCGAAAAAAGAGGGTTTCTGGCGGGAAAAACTCGGTAT**<br>**TTCTTTTTCCTTCAAATGGTTATAGGTTTTCGGAGCTA**GTTCACTGCCGTGTAGGCAGCTAAG<br>AAAATCAGCCGGACGTTGTAGTAGTCGAGCGCGGT |
| CRISPR2 template with native promoter (1 nt…783 nt, from TSS-shown in bold, repeat sequence shown in red, spacer 1 shown in blue, 3 point nucleotides mutation shown in green) | TACAGGCCCTGGAAACGCCGGACAGCTCCCCCGGACATTTCCGCAGGCTCTCTCAGAAGCCCGG<br>GAATTGGATAAAAACCCAGTAGGAGGATGATGGAGG**GGCCGGCAAATCCATCCCGCAGGGAC**<br>**AGGACCGAACGGCACTGCCCTCTATTTTCTGAGGGCAAAAAAGACGCGGGCCGCAGGTG**<br>**GTGGAACACCTACGACCCGCTAACCACAAACCAGCTTATGAAGGAGCTGACCCATGGCTA**<br>**AGCCGAATGATACGTCCAACCCCGAGGTAACAGAATCGTCCCCCCGCTTCGTCCCGCTGC**<br>**TCCCCACCTCCCAGGCCGACATGTGTCCGCGTAGCGCCACCCTGTACGTGGACACCCATG**<br>**CACTCCCCACGCACTTGTTCGACAACGCGAAAGTGCGCTTGAGGTCGGCGATCAACATCC**<br>**TGGCCATGACCCGTTTGCTCGATGAGAAAGAAGAGCCCGGCGGCACCCTGGCCTACATCA**<br>**ACGGTGCGTTGCTGTCGCTGCTCAGCGATGCCTTGTCCGAAATCGAGGCGGGCCACCCGA**<br>**GCCTGTAGCGCCGCTCCCTGGCGCGACGCCCCCGGCCTGGCCGGGGGCTAACACGCATC**<br>**GCCCATCAAAAGACCTTTCGCGCCCGAACGGCACGGTTGATCGCCGTCCCGGTCCTCGCG**<br>**AAACGGCCGCCAATTGCCCGAAGCTTCCGACCCTTTTTTCGGACGATTTCTTACGCCCTTA**<br>**TAAATCAGCAAGTTACGAGACCTCGAAAAAAGAGGGTTTCTGGCGGGAAAAACTCGGTAT**<br>**TTCTTTTTCCTTCAAATGGTTATAGGTTTTCGGAGCTA**GTTCACTGCCGTGTAGGCAGCTAAG<br>AAAATCAGCCGGACGTTGTAGTAGTCGAGCGCGGT |
| T7-PhrS (T7 promoter is underlined) | TAATACGACTCACTATAGGATCGAGCAACACCCAACCGGCAACTGGAGGCCATCAACATGTTC<br>ATCGACGAAGTGGTTCTCGCAGGGATTCTTACAGTAGGCCTCATGGTCGCTTTCTTCGGTGGGG<br>TCGGTTACTTCATCTGGAAGGATTCCCATAGCCGCAAAGGCTGATCCTCCCGGATACACAGAGC<br>ACGCAAGGCACTTAGGGCGACTTCGGTCGCCCGTTTTTTTT |
