# Supplementary material for: High-throughput screen reveals sRNAs regulating crRNA biogenesis by targeting CRISPR leader to repress Rho termination
Source: Nat Commun. 2019 Aug 19;10:3728. doi: 10.1038/s41467-019-11695-8 (PMC6700203; doi:10.1038/s41467-019-11695-8)
Supplement: Supplementary file 3 — Description of Additional Supplementary Files [file 41467_2019_11695_MOESM3_ESM.pdf]

Description of Additional Supplementary Files

File name: Supplementary Data 1.
Description: Intergenic sRNA candidates and annotated sRNA in Pseudomonas aeruginosa binding to CRISPR loci by T4 RNA Ligase in vivo. "1" represents sRNA-containing chimaeras; "0" represents no sRNA chimaeras.

File name: Supplementary Data 2.
Description: Bacterial strains, phage and plasmid used in this study.

File name: Supplementary Data 3.
Description: Primer Sequences for library screening.
